# Synthesis, Biological Evaluation, and Mode of Action of *Pulsatilla* Saponin D Derivatives as Promising Anticancer Agents

**DOI:** 10.3389/fphar.2019.01208

**Published:** 2019-10-15

**Authors:** Yuanying Fang, Daoyong Hu, Huilan Li, Jianguo Hu, Yanhua Liu, Zhifeng Li, Guoliang Xu, Lanying Chen, Yi Jin, Shilin Yang, Zunhua Yang

**Affiliations:** ^1^National Engineering Research Center for Manufacturing Technology of TCM Solid Preparation, Jiangxi University of Traditional Chinese Medicine, Nanchang, China; ^2^The Key Laboratory of Oral Biomedicine, The Affiliated Stomatological Hospital of Nanchang University, Nanchang, China; ^3^Research Center for Differentiation and Development of Basic Theory of Traditional Chinese Medicine, Jiangxi University of Traditional Chinese Medicine, Nanchang, China; ^4^College of Pharmacy, Jiangxi University of Traditional Chinese Medicine, Nanchang, China

**Keywords:** *Pulsatilla* saponin D, synthesis, antiproliferative activity, acute toxicity, apoptosis

## Abstract

A series of ester and amide derivatives of triterpenoid saponin *Pulsatilla* saponin D (PSD) were designed, synthesized, and evaluated for their antiproliferative activity. Compounds 1 and 6 displayed 1.7–8.3 times more potent cytotoxicity (IC_50_ = 1.2–4.7 and 1.7–4.5 μM, respectively) against five human tumor cell lines (SMMC-7721, MCF-7, NCI-H460, A549, and HCT-116) *in vitro* and lower acute toxicity to mice *in vivo* than did PSD. Furthermore, compound 6 was observed to show potent tumor growth inhibition against mice H22 hepatocellular cells (49.8% at 20 mg/kg) and induce cell cycle at G_1_ phase and apoptosis in HCT-116 cells.

## Introduction

The *Pulsatilla chinensis* (Bunge) Regel, a traditional Chinese herb, is used for “blood cooling,” stopping dysentery, and detoxification (Chen et al., 2015; Liu et al., 2013; Jiang et al., 2009; Fang et al., 2016). A series of triterpenoid saponins were isolated from the plant and reported to possess various biological activities such as anticancer, anti-inflammatory, antiviral and antibacterial, and so on (Liang et al., 2011; Ye et al., 2014; Guan et al., 2015; Acebey-Castellon et al., 2011). In our efforts to discover anticancer agents, we focus on the modification of natural product as an effective and powerful strategy, which is also a promising approach to develop new lead compounds (Che et al., 2014).

**Figure 1:**
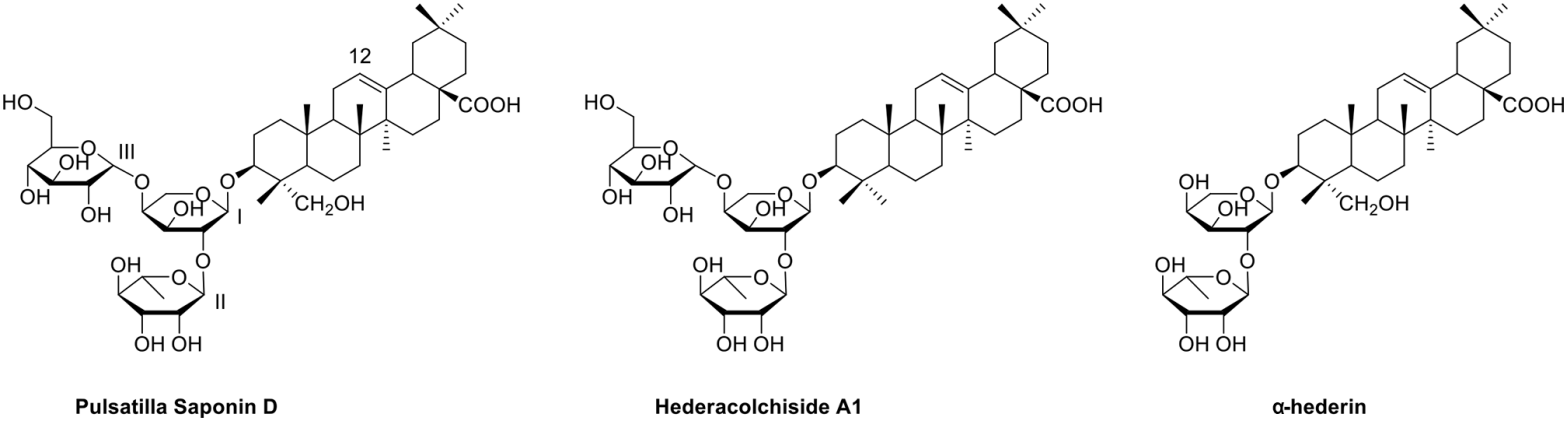
The structures of three triterpenoid saponins.

Among these triterpenoid saponins, several abundant components including hederacolchiside A_1_, *Pulsatilla* saponin D (PSD), and α-hederin (**Figure 1**) exhibited potent antitumor activities *in vitro* and *in vivo* (Chen et al., 2018; Zhang et al., 2015; Shu et al., 2013; Xu et al., 2012; Yan et al., 2008; Kim et al., 2013). However, due to the hemolytic toxicity of triterpenoid saponins, it is impossible to develop them as clinical medicines directly. We have previously investigated hederacolchiside A_1_ derivatives bearing furoxan fragment as a nitric oxide donor against solid tumor in animal models (Fang et al., 2017). Nevertheless, since the isolation of hederacolchiside A_1_ was in a lower yield than PSD, it was difficult to obtain the material, which limited its modification.

In our attempts to discover the valuable anticancer agents, PSD was chosen as the lead compound. PSD (hederagenin 3-O-a-L-rhamnopyranosyl-(1/2)-[b-D-glucopyranosyl-(1/4)]-a-L-arabinopyranoside), also named SB365 in the literature, was isolated from *P. chinensis* (Bunge) Regel in our department and showed excellent bioactivity (Kim et al., 2004; Bang et al., 2005). A structure–activity relationship study indicated that the 28-COOH is the moiety causing hemolytic toxicity, so modification of PSD was conducted by converting it to ester or amide bearing various functional groups such as alkyl, amino, halogen, hydroxyl, and carboxyl groups. We would see whether these moieties were beneficial for enhancement of antitumor activity and lowering of toxicity. In addition, we envisaged that the shelter of carboxyl group would significantly improve antiproliferative activities and hemolysis issue. Herein we presented the synthesis, biological evaluation, and mode of action of PSD derivatives as antitumor agents.

## Results and Discussion

### Chemistry

**Scheme 1:**
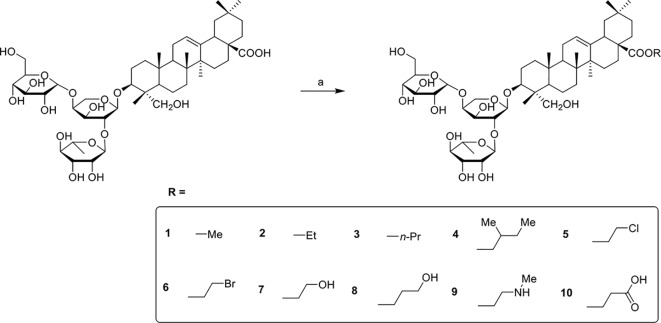
Reagents and conditions: (a) R-I, K_2_CO_3_, DMF, R.T., 12h or R-Br, K_2_CO_3_, DMF, 50°C, 12h or R-Cl, K_2_CO_3_, DMF, NaI, 50°C, 12h.

**Scheme 2:**
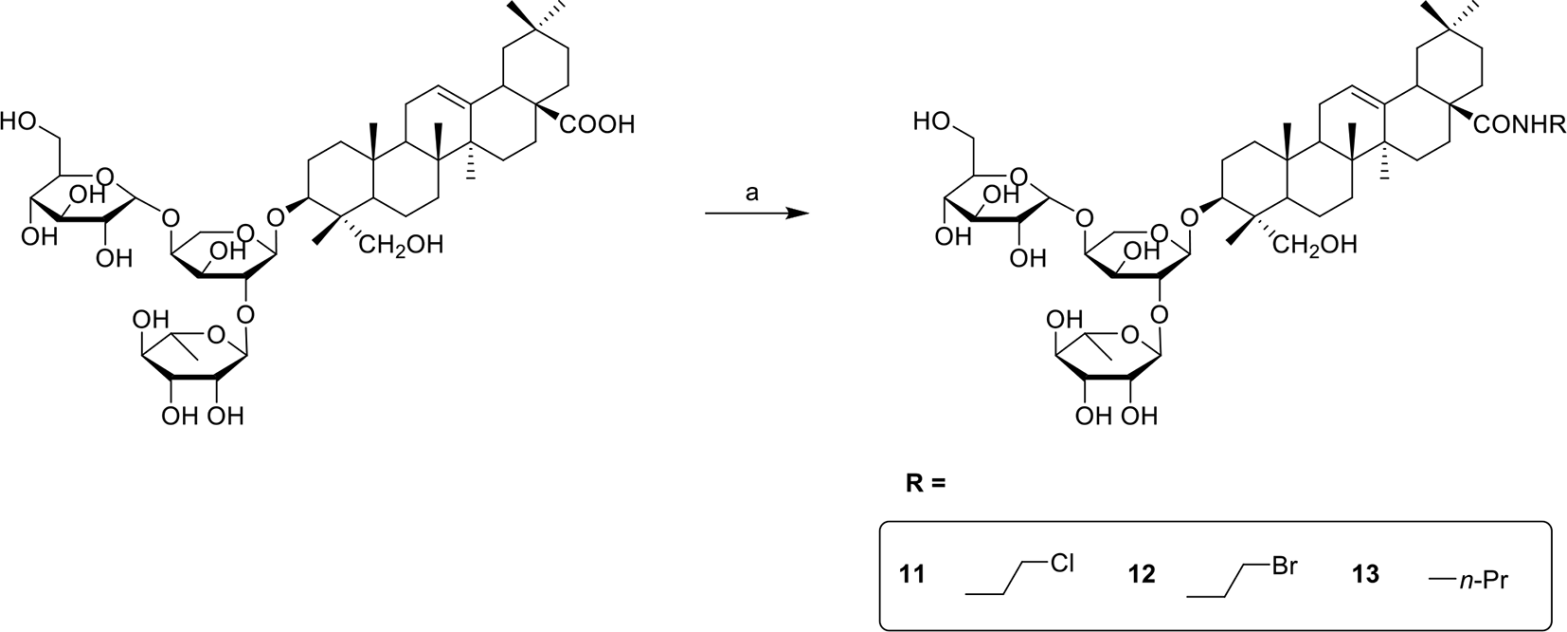
Reagents and conditions: (a) NH_2_-R, EDCI, HOBt, DIPEA, DMF, R.T., 12h.

PSD derivatives were generated from the natural product PSD, following the procedures and conditions as shown in **Schemes 1** and **2**. Esterification of PSD with iodomethane, iodoethane, or 1-iodopropane in the presence of K_2_CO_3_/DMF at room temperature afforded esters 1, 2, and 3, respectively, in good yields. Reaction of PSD with dichloroethane in the presence of K_2_CO_3_/NaI/DMF at 50°C provided chlorinated ester 5 in 52%. Condensation of PSD with bromo-substituted materials in the presence of K_2_CO_3_/DMF at 50°C yielded substituted esters 4 and 6–10, respectively. The amides 11–13 were obtained via condensation of PSD with amines in the presence of EDCI/HOBt/DIPEA/DMF at room temperature.

### Antiproliferative Activity

Compounds 1–13 were evaluated for their antiproliferative activities against SMMC-7721, MCF-7, NCI-H460, A549, and HCT-116 human cancer cell lines using the MTT assay. PSD was selected as a positive control, and the cytotoxicity assay results are illustrated in **Table 1**. Compounds 1 and 6, methyl ester and β-bromoethyl ester of PSD, exhibited 1.7–8.3 times more potent antiproliferative activities than did PSD against all five cancer cell lines. The ethyl ester 2 and β-chloroethyl ester 5 displayed stronger cytotoxicity than did PSD against the four cell lines MCF-7, NCI-H460, A549, and HCT-116 with IC_50_ less than 10 μM, and similar activities to those of PSD against SMMC-7721. In general, the halogen substitution and ester derivatives were favorable to the activities compared with hydroxyl and amide, respectively. However, the β-carboxylic ethyl ester 10 also showed superior cytotoxicity to PSD against the three cell lines MCF-7, NCI-H460, and HCT-116.

**Table 1:** Cytotoxicity of PSD derivatives against 5 human cancer cell lines after 48 h incubation.

Compound	IC_50_^a^ (μM)				
	SMMC-772^b^	MCF-7^c^	NCI-H460^d^	A549^e^	HCT-116^f^
1	2.6 ± 0.4	3.9 ± 0.2	1.8 ± 0.3	1.2 ± 0.1	4.7 ± 0.7
2	4.9 ± 0.7	6.2 ± 1.1	5.5 ± 1.0	3.8 ± 0.9	8.1 ± 1.5
3	8.4 ± 1.3	13.2 ± 2.1	14.7 ± 2.0	10.5 ± 1.6	14.9 ± 1.8
4	18.0 ± 2.7	23.5 ± 3.0	21.9 ± 3.5	27.7 ± 3.6	30.2 ± 4.1
5	5.5 ± 0.9	8.1 ± 1.2	3.9 ± 0.9	6.2 ± 1.0	3.5 ± 0.7
6	2.4 ± 0.3	4.5 ± 0.7	3.2 ± 0.2	3.7 ± 0.4	1.7 ± 0.4
7	20.8 ± 3.5	33.2 ± 4.7	27.0 ± 3.8	ND^g^	24.6 ± 3.1
8	22.5 ± 2.8	41.1 ± 6.2	33.1 ± 4.8	ND	39.7 ± 5.1
9	13.8 ± 1.7	20.2 ± 3.5	17.3 ± 2.4	ND	23.8 ± 3.0
10	10.4 ± 1.7	5.2 ± 0.9	3.7 ± 0.8	ND	6.8 ± 1.0
11	10.5 ± 2.3	21.1 ± 3.5	18.3 ± 3.0	30.5 ± 4.1	25.7 ± 3.2
12	8.8 ± 1.2	13.2 ± 2.7	10.0 ± 1.8	12.7 ± 2.2	13.9 ± 1.6
13	9.4 ± 1.4	10.7 ± 1.5	38.7 ± 3.9	ND	14.6 ± 1.8
PSD	4.5 ± 0.9	3.7 ± 0.2	10.8 ± 1.5	7.9 ± 1.1	14.1 ± 1.4

a Concentration inhibiting fifty percent of cell growth for 48 h exposure period of tested samples. Assay was done in triplicate.

b Human hepatocellular cell line.

c Human breast cell line.

d Human large cell lung cancer cell line.

e Human lung cancer cell line.

f Human colon cancer cell line.

g Not determined.

### Mice Acute Toxicity

PSD derivatives **1** and **6** exhibited potent antitumor activities on five human cancer cell lines (IC_50_ = 1.2–4.7 and 1.7–4.5 μM, respectively) and were selected to identify the mice acute toxicities compared with PSD. Due to the poor solubility, these three compounds were made in micelle formation with methoxyl poly(ethylene glycol)-poly(lactide) copolymer (mPEG-PLA; molecular weight, 4000) at 1.0-mg/ml concentration of PSD and at 2.4-mg/ml concentration of **1** and **6**. The mice were injected with the solutions *via* the caudal vein at a dose of 0.1 ml/10 g/d for 7 days and continued to be under observation for another 7 days without injection. As shown in **Table 2**, the tolerance dose of natural product PSD was less than 10 mg/kg, and 12 mice were all dead in 1 h after injection. The tolerance doses of **1** and **6** were both higher than 168 mg/kg, and no mouse was dead in 14 days. The results indicated that **1** and **6** declined the acute toxicity greatly and supplied an evidence to design the suitable dose for tumor inhibition rate trial.

**Table 2:** Mice acute toxicity of compound 1 and 6.

Compound	Number of mice	Tolerance dose (mg/kg)	Death number	Death time
**1**	12	>68	0	> 14 days
**6**	12	>168	0	> 14 days
**PSD**	12	<10	12	< 1 h

### Tumor Growth Inhibition Study *In Vivo*

Compound **6** was observed to exhibit significant and consistent cytotoxicity in five human cancer cell lines without apparent mice acute toxicity. The effect of compound **6** on tumor growth inhibition was evaluated using H22 hepatocellular carcinoma model in male mice with PSD as a positive control. Compounds 6 and PSD were made in micelle with mPEG-PLA at concentrations of 2.4 and 1.0 mg/ml, respectively. Then compound 6 was injected via the caudal vein at doses of 10 and 20 mg/kg/d for 14 days, respectively; PSD was injected at a dose of 6 mg/kg/3 d five times. The results are illustrated in **Table 3**. Treatment with 6 at 10 mg/kg significantly inhibited the growth of H22 tumor in 42.8%, but treatment with 6 at 20 mg/kg enhanced the inhibitory effect on the growth of H22 tumor (49.8%), which was almost equal to the treatment of PSD (50.2%). Remarkably, no mice were dead after treatment with 6 in 14 days, which indicated that derivative 6 could reduce the toxicity greatly.

**Table 3:** *In vivo* H22 xenograft studies of compound 6.

Compound	Number of mice	Dose(mg/kg)	Death number	Tumor weights(g)	Tumor inhibition rates (%)
Model group	13	–	0	2.15 ± 1.54	
6	13	10	0	1.23 ± 1.09*	42.8
6	13	20	0	1.08 ± 1.11*	49.8
PSD	13	6	2	1.07 ± 0.52**	50.2

Control with model group, *P < 0.05, **P < 0.01.

### Cell Cycle Study

**Figure 2:**
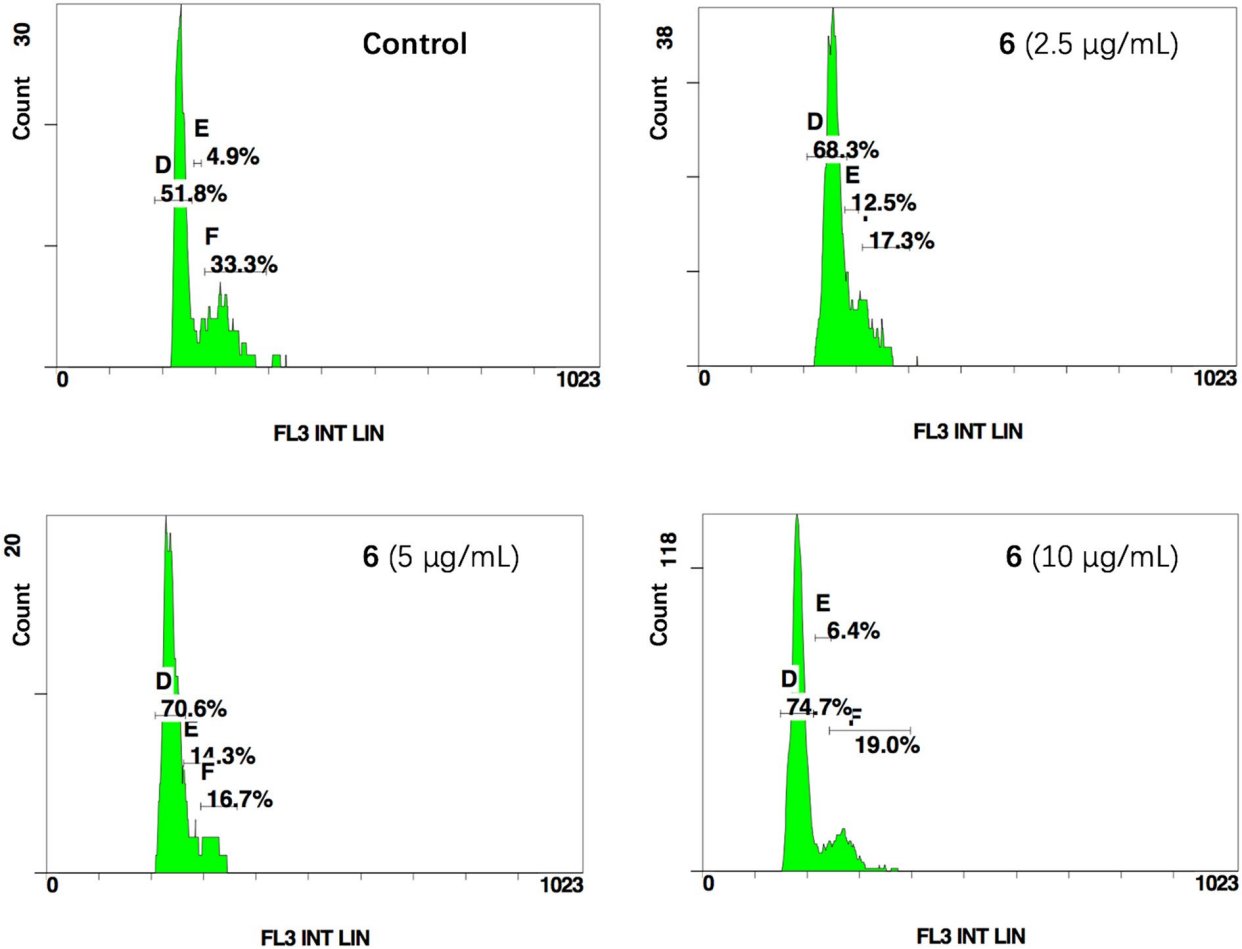
Compound 6 induced G_1_ cycle arrest in HCT-116. D, E and F represent G_1_, S and G_2_ phase.

To elucidate the relationship between the mechanism of cell growth suppression and cell cycle arrest, cell cycle distribution on HCT-116 cells by treating with compound 6 was investigated. As shown in **Figure 2**, the cells of the control group in G_1_, S, and G_2_ phases accounted for 51.8%, 4.9%, and 33.3%, respectively. After cells were treated with 6 at concentrations of 2.5, 5 and 10 μg/ml, the cell percentages of G_1_ phase increased to 68.3%, 70.6%, and 74.7%, respectively. These results indicated that compounds 6 could inhibit tumor cell proliferation by blocking the cell cycle at G_1_ phase in a concentration-dependent manner.

### Apoptosis Induction

**Figure 3:**
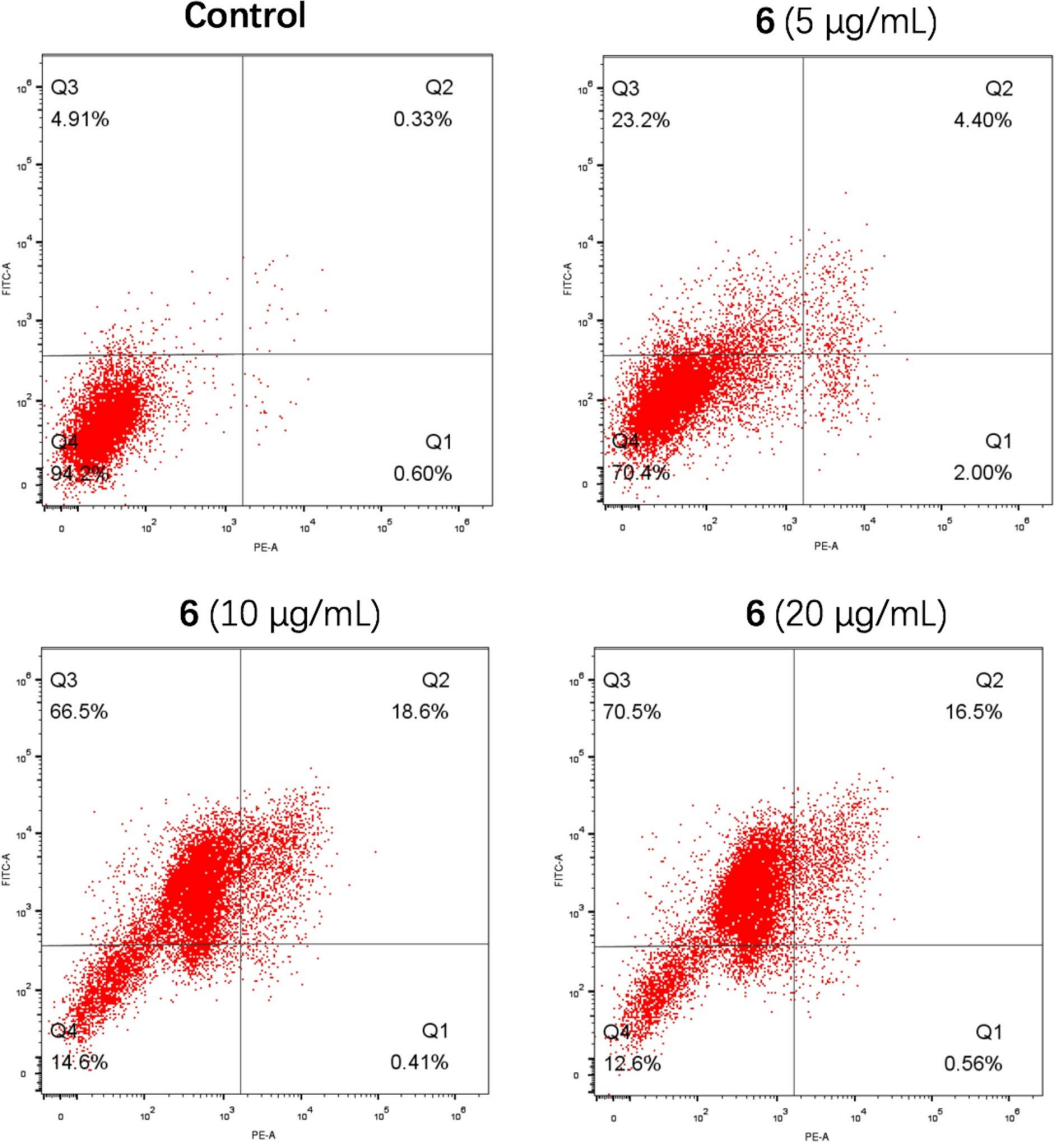
Compound 6 induced apoptosis in HCT-116. Q2 and Q3 represent late and early apoptosis.

Activation of apoptosis is always involved in the mechanism of action of antiproliferative agents. An Annexin V–FITC and propidium iodide (PI) double-staining assay was used to evaluate the effect of compound 6 on the induction of apoptosis. The HCT-116 cells were treated with three concentrations of 6 (5, 10, and 20 μg/ml) for 48 h, and the apoptotic cells were determined. As shown in **Figure 3**, the ratios of early and late apoptotic cells were 27.6%, 85.1%, and 87.0% at the respective concentrations, which indicated that compound 6 induced apoptosis in a concentration-dependent manner also.

## Statistical Methods

*T*-test was used to evaluate the statistical differences between samples. Differences were considered to be significant when *p* < 0.05.

## Conclusion

In summary, 13 PSD derivatives were designed, synthesized, and evaluated for their antiproliferative activities. Among them, compounds 1 and 6 were revealed to exhibit the most potent cytotoxicity against all five cancer cell lines with greatly reduced mice acute toxicity. Remarkably, compound 6 displayed 8.3 times more potent cytotoxicity against HCT-116 (IC_50_ = 1.7 μM) than did PSD and equal potent tumor inhibition rate *in vivo* (49.8%). The mode of action study revealed that compound 6 caused cell cycle arrest of G_1_ phase and induced apoptosis in HCT-116 cells. These results encourage us to develop a preclinical candidate as an anticancer agent from natural products. The follow-up studies on pharmacokinetics of active compounds are in progress, and the results will be reported in due course.

## Materials and Methods

### Chemistry

All starting materials were obtained from commercial suppliers and used without further purification. ^1^H-NMR and ^13^C-NMR spectra were recorded on a Bruker AVANCE III HD 600 (600 Hz) spectrometer. Chemical shifts are reported in parts per million downfield relative to tetramethylsilane as an internal standard. Peak splitting patterns are abbreviated as s (singlet), br s (broad singlet), d (doublet), t (triplet), dd (doublet of doublet), and m (multiplet). Mass spectra were recorded on a Thermo Fisher (LCQ Fleet). High-resolution mass spectrometry (HRMS) spectra were recorded on an AB SCIEX (Triple TOF 5600+). Thin-layer chromatography was performed on silica F254 purchased from the Branch of Qingdao Haiyang Chemical Co. and detected by UV light at 254 and 365 nm or by charring with sulfuric acid. Column chromatography was performed on silica gel column (200–300 mesh; Branch of Qingdao Haiyang Chemical Co.).

### General Procedure to Synthesize Compounds 1-3

To a solution of PSD (100 mg, 0.11 mmol) in DMF (3 ml) was added K_2_CO_3_ (45 mg, 0.33 mmol) and alkyl iodide (0.33 mmol), respectively. The reaction was stirred at room temperature overnight. Then the mixture was poured into ice water and extracted with ethyl acetate (25 ml × 2). The organic phase was combined, washed with saturated brine (15 ml × 2), dried over anhydrous MgSO_4_, and filtered. The filtrate was evaporated to give the crude product, which was purified by column chromatography (dichloromethane methanol mixture as eluent) to afford the target compounds.

#### Methyl Ester of PSD (1)

White solid, 75% yield. ^1^H-NMR (600 Hz, MeOD): δ 5.27 (t, *J* = 3.4 Hz, 1H, H-12), 5.23 (s, 1H, H-1^II^), 4.51 (d, *J* = 6.1 Hz, 1H, H-1^III^), 4.46 (d, *J* = 7.6 Hz, 1H, H-1^I^), 3.64 (s, 3H, COO**CH_3_**), 1.26 (d, *J* = 6.2 Hz, 3H, H-6^II^), 1.19 (s, 3H), 0.99 (s, 3H), 0.96 (s, 3H), 0.93 (s, 3H), 0.77 (s, 3H), 0.73 (s, 3H). HRMS–time-of-flight mass spectrometry (TOF; m/z) calcd for C_48_H_78_O_17_ [M + H]^+^: 927.5317, found, 927.5329.

#### Ethyl Ester of PSD (2)

White solid, 71% yield. ^1^H-NMR (600 MHz, MeOD) δ 5.25 (t, *J* = 3.3 Hz, 1H, H-12), 5.22 (s, 1H, H-1^II^), 4.49 (d, *J* = 6.1 Hz, 1H, H-1^III^), 4.44 (d, *J* = 7.6 Hz, 1H, H-1^I^), 4.11–4.02 (m, 2H, COO**CH_2_**CH_3_), 1.23 (t, *J* = 6.9 Hz, 6H, COOCH_2_ **CH_3_**), 1.18 (s, 3H), 0.98 (s, 3H), 0.94 (s, 3H), 0.91 (s, 3H), 0.77 (s, 3H), 0.71 (s, 3H). HRMS-TOF (m/z) calcd for C_49_H_80_O_17_ [M + H]^+^: 941.5474, found 941.5463.

#### n-Propyl Ester of PSD (3)

White solid, 64% yield. ^1^H-NMR (600 MHz, MeOD) δ 5.27 (t, *J* = 3.5 Hz, 1H, H-12), 5.23 (d, *J* = 1.4 Hz, 1H, H-1^II^), 4.52 (d, *J* = 6.1 Hz, 1H, H-1^III^), 4.46 (d, *J* = 7.6 Hz, 1H, H-1^I^), 4.04–3.96 (m, 2H, COO**CH** **_2_**CH_2_CH_3_), 1.70 (m, 2H, COOCH_2_ **CH_2_**CH_3_), 1.26 (d, *J* = 6.2 Hz, 3H, H-6^II^), 1.18 (s, 3H), 0.99 (m, 3H, COOCH_2_CH_2_ **CH_3_**), 0.98 (s, 3H), 0.95 (s, 3H), 0.93 (s, 3H), 0.78 (s, 3H), 0.73 (s, 3H). HRMS-TOF (m/z) calcd for C_50_H_82_O_17_ [M + H]^+^: 955.5630, found 933.5644.

### General Procedure to Synthesize Compounds 4-10

To a solution of PSD (100 mg, 0.11 mmol) in DMF (3 ml) was added K_2_CO_3_ (45 mg, 0.33 mmol) and bromide (0.33 mmol; or 0.33 mmol of dichloroethane plus catalytic amount of NaI for **5**), respectively. The reaction was stirred at 50°C overnight. Then the mixture was poured into ice water and extracted with ethyl acetate (25 ml × 2). The organic phase was combined, washed with saturated brine (15 ml × 2), dried over anhydrous MgSO_4_, and filtered. The filtrate was evaporated to give the crude product, which was purified by column chromatography (dichloromethane methanol mixture as eluent) to afford the target compounds.

#### **β**-Methyl Butyl Ester of PSD (4)

White solid, 72% yield. ^1^H-NMR (600 MHz, MeOD) δ 5.27 (t, *J* = 3.5 Hz, 1H, H-12), 5.23 (d, *J* = 1.3 Hz, 1H, H-1^II^), 4.51 (d, *J* = 6.1 Hz, 1H, H-1^III^), 4.46 (d, *J* = 7.6 Hz, 1H, H-1^I^), 3.92–3.89 (m, 2H, COO**CH_2_**CH(CH_3_)CH_2_CH_3_), 1.52–1.47 (m, 2H, COOCH_2_CH(CH_3_)**CH_2_**CH_3_), 1.26 (d, *J* = 6.2, 3H, H-6^II^), 1.20 (s, 3H), 1.03 (m, 1H, COOCH_2_ **CH**(CH_3_)CH_2_CH_3_), 0.99 (s, 3H), 0.98 (d, *J* = 6.8 Hz, 3H, COOCH_2_CH(**CH_3_**)CH_2_CH_3_), 0.96 (s, 3H), 0.94 (m, 6H, COOCH_2_CH(CH_3_)CH_2_ **CH_3_**), 0.77 (s, 3H), 0.73 (s, 3H). HRMS-TOF (m/z) calcd for C_52_H_87_O_17_ [M + H]^+^: 983.5943, found 983.5935.

#### **β**-Chloroethyl Ester of PSD (5)

White solid, 58% yield. ^1^H-NMR (600 MHz, MeOD) δ 5.29 (t, *J* = 3.5 Hz, 1H, H-12), 5.23 (d, *J* = 1.4 Hz, 1H, H-1^II^), 4.51 (d, *J* = 6.1 Hz, 1H, H-1^III^), 4.46 (d, *J* = 7.6 Hz, 1H, H-1^I^), 4.35–4.29 (m, 1H, COO**CH_2_**CH_2_Cl), 4.23 (m, 1H, COO**CH_2_**CH_2_Cl), 3.74 (m, 2H, COOCH_2_ **CH_2_**Cl), 1.26 (d, *J* = 6.2 Hz, 3H), 1.20 (s, 3H), 0.99 (s, 3H), 0.97 (s, 3H), 0.94 (s, 3H), 0.79 (s, 3H), 0.73 (s, 3H). ^13^C-NMR (150Hz, MeOD) δ 177.6, 143.4, 122.6, 104.7, 103.2, 100.5, 80.8, 78.4, 76.7, 76.5, 75.5, 73.9, 72.6, 72.5, 70.7, 70.6, 69.9, 65.4, 64.1, 63.7, 63.1, 61.2, 46.8, 46.7, 45.6, 42.5, 41.6, 41.5, 41.4, 39.2, 38.3, 36.2, 35.5, 33.4, 32.3, 32.1, 32.0, 30.2, 27.4, 25.1, 25.0, 23.1, 22.6, 22.5, 17.4, 16.6, 16.4, 15.0, 12.4. HRMS-TOF (m/z) calcd for C_49_H_79_ClO_17_ [M + H]^+^: 975.5084, found 975.5106.

#### **β**-Bromoethyl Ester of PSD (6)

White solid, 73% yield. ^1^H-NMR (600 MHz, MeOD) δ 5.29 (t, *J* = 3.6 Hz, 1H, H-12), 5.23 (s, 1H, H-1^II^), 4.51 (d, *J* = 6.1 Hz, 1H, H-1^III^), 4.45 (t, *J* = 6.9 Hz, 1H, H-1^I^), 4.40–4.35 (m, 1H, COO**CH_2_**CH_2_Br), 4.32–4.27 (m, 1H, COO**CH_2_**CH_2_Br), 3.60-3.58 (m, 2H, COOCH_2_ **CH_2_**Br), 1.25 (d, *J* = 6.1 Hz, 3H), 1.19 (s, 3H), 0.99 (s, 3H), 0.96 (s, 3H), 0.93 (s, 3H), 0.79 (s, 3H), 0.73 (s, 3H). ^13^C-NMR (150Hz, MeOD) δ 177.5, 143.4, 122.7, 104.7, 103.2, 100.5, 80.8, 78.4, 76.7, 76.5, 75.5, 73.9, 72.6, 72.5, 70.7, 70.6, 69.9, 68.7, 64.0, 63.7, 63.2, 61.2, 46.8, 46.7, 45.7, 42.6, 41.5, 41.3, 39.3, 38.3, 36.2, 35.6, 33.4, 32.3, 32.1, 32.0, 30.2, 28.9, 27.4, 25.1, 25.0, 23.1, 22.6, 22.5, 17.4, 16.6, 16.5, 15.0, 12.4. HRMS-TOF (m/z) calcd for C_49_H_79_BrO_17_ [M + H]^+^: 1019.4579, found 1019.4585.

#### **β**-Hydroxyethyl Ester of PSD (7)

White solid, 70% yield. ^1^H-NMR (600 MHz, MeOD) δ 5.27 (t, *J* = 3.5 Hz, 1H, H-12), 5.21 (d, *J* = 1.4 Hz, 1H, H-1^II^), 4.49 (d, *J* = 6.1 Hz, 1H, H-1^III^), 4.44 (d, *J* = 7.6 Hz, 1H, H-1^I^), 4.13 (m, 2H, COO**CH_2_**CH_2_OH), 3.71 (m, 2H, COOCH_2_ **CH_2_**OH), 1.24 (d, *J* = 6.2 Hz, 3H), 1.18 (s, 3H), 0.97 (s, 3H), 0.94 (s, 3H), 0.91 (s, 3H), 0.77 (s, 3H), 0.71 (s, 3H). HRMS-TOF (m/z) calcd for C_49_H_80_O_18_ [M + H]^+^: 957.5423, found 957.5433.

#### **γ**-Hydroxypropyl Ester of PSD (8)

White solid, 55% yield. ^1^H-NMR (600 MHz, MeOD) δ 5.28 (t, *J* = 3.6 Hz, 1H, H-12), 5.20 (d, *J* = 1.3 Hz, 1H, H-1^II^), 4.48 (d, *J* = 6.2 Hz, 1H, H-1^III^), 4.42 (d, *J* = 7.6 Hz, 1H, H-1^I^), 4.18–4.14 (m, 2H, COO**CH_2_**CH_2_CH_2_OH), 3.83–3.69 (m, 2H, COOCH_2_CH_2_ **CH_2_**OH), 1.88–1.83 (m, 2H, COOCH_2_ **CH_2_**CH_2_OH), 1.25 (d, *J* = 6.2 Hz, 3H), 1.15 (s, 3H), 0.98 (s, 3H), 0.95 (s, 3H), 0.91 (s, 3H), 0.76 (s, 3H), 0.71 (s, 3H). HRMS-TOF (m/z) calcd for C_50_H_82_O_18_ [M + H]^+^: 971.5579, found 971.5595.

#### **β**-Methylaminoethyl Ester of PSD (9)

White solid, 51% yield. ^1^H-NMR (600 MHz, MeOD) δ 5.26 (s, 1H, H-12), 5.23 (s, 1H, H-1^II^), 4.52 (d, *J* = 6.1 Hz, 1H, H-1^III^), 4.46 (d, *J* = 7.6 Hz, 1H, H-1^I^), 4.36–4.31 (m, 2H, COO**CH_2_**CH_2_NHCH_3_), 3.38 (m, 2H, COOCH_2_ **CH_2_**NHCH_3_), 3.37 (m, 3H, COOCH_2_CH_2_NH**CH_3_**), 1.26 (d, *J* = 6.2 Hz, 3H), 1.20 (s, 3H), 1.00 (s, 3H), 0.96 (s, 3H), 0.93 (s, 3H), 0.84 (s, 3H), 0.73 (s, 3H). HRMS-TOF (m/z) calcd for C_50_H_83_NO_17_ [M + H]^+^: 970.5739, found 970.5753.

#### **β**-Carboxyethyl Ester of PSD (10)

White solid, 42% yield. ^1^H-NMR (600 MHz, MeOD) δ 5.26 (s, 1H, H-12), 5.24 (d, *J* = 1.3 Hz, 1H, H-1^II^), 4.52 (d, *J* = 6.1 Hz, 1H, H-1^III^), 4.47 (d, *J* = 7.7 Hz, 1H, H-1^I^), 4.39–4.34 (m, 2H, COO**CH_2_**CH_2_COOH), 2.67–2.57 (m, 2H, COOCH_2_ **CH_2_**COOH), 1.26 (d, *J* = 6.2 Hz, 3H), 1.19 (s, 3H), 0.99 (s, 3H), 0.96 (s, 3H), 0.93 (s, 3H), 0.84 (s, 3H), 0.73 (s, 3H). HRMS-TOF (m/z) calcd for C_50_H_80_O_19_ [M + H]^+^: 985.5372, found 985.5386.

### General Procedure to Synthesize Compounds 11-13

To a solution of PSD (100 mg, 0.11 mmol) in DMF (3 ml) was added EDCI (42 mg, 0.22 mmol), HOBt (30 mg, 0.22 mmol), DIPEA (43 mg, 0.33 mmol), and aliphatic amine (0.33 mmol), respectively. The reaction was stirred at room temperature overnight. Then the mixture was poured into ice water and extracted with ethyl acetate (25 ml × 2). The organic phase was combined, washed with saturated brine (15 ml × 2), dried over anhydrous MgSO_4_, and filtered. The filtrate was evaporated to give the crude product, which was purified by column chromatography (dichloromethane methanol mixture as eluent) to afford the target compounds.

#### **β**-Chloroethyl Amide of PSD (11)

White solid, 67% yield. ^1^H-NMR (600 MHz, MeOD) δ 5.38 (t, *J* = 3.3 Hz, 1H, H-12), 5.23 (s, 1H, H-1^II^), 4.72–4.66 (m, 1H, CONH**CH_2_**CH_2_Cl), 4.66–4.60 (m, 1H, CONH**CH_2_**CH_2_Cl), 4.51 (d, *J* = 6.1 Hz, 1H, H-1^III^), 4.46 (d, *J* = 7.7 Hz, 1H, H-1^I^), 3.65–3.58 (m, 2H, CONHCH_2_ **CH_2_**Cl), 1.26 (d, *J* = 6.2 Hz, 3H), 1.20 (s, 3H), 0.97 (s, 3H), 0.94–0.93 (m, 3H), 0.93 (s, 3H), 0.74 (s, 3H), 0.71 (s, 3H). HRMS-TOF (m/z) calcd for C_49_H_80_ClNO_16_ [M + H]^+^: 974.5244, found 974.5230.

#### **β**-Bromoethyl Amide of PSD (12)

White solid, 58% yield. ^1^H-NMR (600 MHz, MeOD) δ 5.37 (t, *J* = 3.4 Hz, 1H, H-12), 5.23 (d, *J* = 1.3 Hz, 1H, H-1^II^), 4.73–4.66 (m, 1H, CONH**CH_2_**CH_2_Br), 4.63 (m, 1H, CONH**CH_2_**CH_2_Br), 4.51 (d, *J* = 6.1 Hz, 1H, H-1^III^), 4.46 (d, *J* = 7.7 Hz, 1H, H-1^I^), 3.61 (m, 2H, CONHCH_2_**CH_2_**Br), 1.26 (d, *J* = 6.2 Hz, 3H), 1.20 (s, 3H), 0.97 (s, 3H), 0.94 (s, 3H), 0.93 (s, 3H), 0.74 (s, 3H), 0.71 (s, 3H). HRMS-TOF (m/z) calcd for C_49_H_80_BrNO_16_ [M + H]^+^: 1018.4739, found 1018.4755.

#### n-Propyl Amide of PSD (13)

White solid, 70% yield. ^1^H-NMR (600 MHz, MeOD) δ 5.37 (t, J = 3.3 Hz, 1H, H-12), 5.23 (d, *J* = 1.3 Hz, 1H, H-1^II^), 4.52 (d, *J* = 6.1 Hz, 1H, H-1^III^), 4.46 (d, *J* = 7.6 Hz, 1H, H-1^I^), 3.19 (m, 1H, CONH**CH_2_**CH_2_CH_3_), 3.04 (m, 1H, CONH**CH_2_**CH_2_CH_3_), 1.52 (m, 2H, CONHCH_2_**CH_2_**CH_3_), 1.26 (d, *J* = 6.2 Hz, 3H), 1.20 (s, 3H), 0.99 (s, 3H), 0.97 (s, 3H), 0.93 (m, 6H, CONHCH_2_CH_2_**CH_3_**), 0.81 (s, 3H), 0.73 (s, 3H). ^13^C-NMR (150 MHz, MeOD) δ 178.9, 144.1, 122.6, 104.7, 103.2, 100.5, 80.8, 78.3, 76.7, 76.5, 75.5, 73.9, 72.6, 72.5, 70.7, 70.6, 69.9, 68.7, 63.7, 63.9, 1, 61.3, 46.8, 46.3, 46.1, 42.6, 41.7, 41.2, 41.1, 39.3, 38.3, 36.2, 33.7, 33.0, 32.2, 31.9, 30.2, 29.4, 27.1, 25.1, 25.0, 23.2, 22.6, 22.5, 22.2, 17.4, 16.6, 16.5, 15.0, 12.4, 10.5. HRMS-TOF (m/z) calcd for C_50_H_83_NO_16_ [M + H]^+^: 954.5790, found 954.5814.

### Mtt Assay

Cell culture and cytotoxicity test: The human cancer cell lines were purchased from Shanghai Institutes for Biological Sciences and maintained in a humidified atmosphere at 37°C in 5% CO_2_. The cells were grown in RPMI-1640 (GIBCO) media containing 10% heat-inactivated fetal bovine serum. Cytotoxicity was determined by the MTT assay according to the manufacturer’s protocol. Briefly, cells were seeded in 96-well microtiter plates at a density of 8 × 10^3^ cells per well. After a 24-h incubation, cells were treated with various concentrations of PSD derivatives, cultured for 48 h. At the end of the treatment period, 20 μl of the MTT (5 mg/ml) reagent was added to each well. After a 4-h incubation at 37°C, the supernatant was aspirated, and formazan crystals were dissolved in 150 μl DMSO for 10 min with gentle agitation. The absorbance per well was measured at 490 nm with a SpectraMax i3 (Molecular Devices Corp.). The assay was done in triplicate. The IC_50_ values were then determined for each compound from a plot of log (drug concentration) versus percentage of loss of viability

### Tumor Growth Inhibition *In Vivo*

Male KM mice with a weight of 18–22 g were inoculated subcutaneously with 5 × 10^6^ H22 cells (purchased from the Chinese Academy of Medical Sciences). After 72 h, the tumor-bearing mice were randomized into four groups. The three test groups were treated *via* caudal vein injection with 6 mg/kg/3 d of PSD five times, or 10 or 20 mg/kg/d of compound 6 for 14 days, respectively. After 24 h of the last administration, the mice were sacrificed, and their tumors were dissected out and weighted.

### Cell Cycle Analysis

HCT-116 cells were plated in 6-well plates and incubated at 37°C for 24 h, then incubated with or without 2.5, 5, and 10 μg/ml of 6, respectively. After 48 h, the cells were centrifuged and fixed in 70% ethanol at 4°C overnight, washed with PBS, treated with 20 μl RNase A (1 mg/ml), and stained with 20 μl PI (1 mg/ml) for 30 min. Cellular DNA content was determined using a flow cytometer (Beckman–Coulter Gallios).

### Apoptosis Analysis

Apoptosis-mediated cell death of tumor cells was examined using an FITC–Annexin V/PI apoptosis detection kit (BD Pharmingen™) according to the manufacturer’s instructions. In brief, 1 × 10^6^ cells were harvested and washed with PBS. They were resuspended in 500 μl of binding buffer, and then 5 μl of FITC–Annexin V and 5 μl of PI were added. Flow cytometric analysis was performed immediately after supravital staining. Data acquisition and analysis were performed with BD FACSCalibur™ flow cytometer. The cells in early stages of apoptosis were Annexin V positive and PI negative, whereas the cells in the late stage of apoptosis were both Annexin V and PI positive.

## Data Availability Statement

All datasets generated for this study are included in the manuscript/supplementary files.

## Ethics Statement

The animal study was reviewed and approved by the experimental animal ethics committee, Jiangxi University of Traditional Chinese Medicine.

## Author Contributions

YF, GX, and ZY designed the project. YF, JH, and YL performed the chemistry experiments. DH, HL, and ZL completed the biology experiments. YF, DH, and ZY analyzed the data and wrote the manuscript. LC, YJ, and SY provided instructions for the project.

## Funding

This work was financially supported by the Jiangxi Provincial Department of Science and Technology (20171BAB205103, 20181BAB215043, 20192ACB21012), Education Department of Jiangxi Province on Science and Technology Project Foundation (GJJ180667), Health and Family Planning Commission of Jiangxi Province (2017A297, 20185523), Jiangxi University of Traditional Chinese Medicine (JXSYLXK-ZHYAO013), and Nanchang Innovative Talent Team (No. [2018]274).

## Conflict of Interest

The authors declare that the research was conducted in the absence of any commercial or ﬁnancial relationships that could be construed as a potential conﬂict of interest.
